# microRNA‐Mediated Regulation of Bone Remodeling: A Brief Review

**DOI:** 10.1002/jbm4.10213

**Published:** 2019-08-07

**Authors:** Jin Liu, Lei Dang, Xiaohao Wu, Dijie Li, Qing Ren, Aiping Lu, Ge Zhang

**Affiliations:** ^1^ Law Sau Fai Institute for Advancing Translational Medicine in Bone & Joint Diseases Hong Kong Baptist University, Hong Kong SAR China; ^2^ School of Life Sciences Northwestern Polytechnical University Xi'an China

**Keywords:** BONE MODELING AND REMODELING, OSTEOBLASTS, OSTEOCLASTS, OSTEOCYTES, MICRO RNA

## Abstract

microRNA (miRNA)‐mediated regulation represents a highly efficient posttranscriptional mechanism for controlling intracellular protein expression. In the past decade, many studies have shown that various miRNAs are involved in regulating bone remodeling by affecting different stages of osteoblastogenesis, osteocytic differentiation, and osteoclastogenesis to govern osteoblastic bone formation and osteoclastic bone resorption. Moreover, miRNAs are recently implicated in mediating the cell‐cell communications among bone cells. This review concentrates on the miRNA‐mediated regulatory mechanisms of osteoblasts, osteoclasts, and osteocytes, and their contribution to bone remodeling. © 2019 The Authors. *JBMR Plus* published by Wiley Periodicals, Inc. on behalf of American Society for Bone and Mineral Research.

## Introduction

Bone is a dynamic organ that grows and adapts its shape and structure by modeling in childhood and undergoing constant remodeling in the whole life. Osteoblasts are bone‐forming cells that govern new bone formation, whereas osteoclasts are bone resorbing cells capable of removing old bone matrix.^1^ The functions of these two types of cells are not only precisely controlled by their distinct intracellular molecular events, but also regulated by the coupling factors during their interaction with each other. The dysregulation of any intracellular event of each cell type or the impairment in their coupling factors will affect bone development and remodeling.^2^

microRNAs (miRNAs) are a class of endogenous, evolutionarily conserved, small long‐noncoding RNAs (generally 20 to 24 nucleotides long) that regulate gene expression at the posttranscriptional level to coordinate a broad spectrum of biological processes.^3^ Mechanistically, miRNAs directly bind to the three prime untranslated region (3′UTR) of messenger RNAs (mRNAs) to block their translation or induce mRNA degradation. The genes encoding the miRNAs are initially transcribed as primary miRNAs (pri‐miRNAs, ~80 nucleotides long) in the nucleus by RNA polymerase II (Pol II), and further cleaved by the ribonuclease II called Drosha or double‐stranded DNA‐binding protein DGCR8 (Di George syndrome critical gene 8), giving rise to precursor miRNAs (pre‐miRNAs, ~70 nucleotides long) with hairpin structures. The pre‐miRNAs are subsequently exported to the cytoplasm by the nucleocytoplasmic shuttler Exportin‐5 in complex with Ran‐GTP, and processed by the endoribonuclease Dicer and the co‐regulator Ago2 to form small double‐stranded miRNAs.^4^, ^5^, ^6^, ^7^ Thereafter, the duplex miRNAs are converted into mature single‐stranded miRNAs (~22 nucleotides long), and incorporated into the RNA‐induced silencing complex (RISC) to target the 3′UTR of mRNAs and mediate gene silencing. The biogenesis of miRNAs is vital for life, because global deletion of either Drosha or Dicer results in early embryonic lethality.^8^ Consistently, the conditional knockout of these key miRNA processing factors in skeletal cells, eg, chondrocytes, osteoblasts, and osteoclasts, respectively, leads to skeletal defects,^9^, ^10^, ^11^, ^12^, ^13^, ^14^ highlighting the crucial role of miRNAs in skeleton development and bone remodeling. Therefore, this review summarizes studies in the past decade focusing on miRNA‐regulatory mechanisms of osteoblast, osteoclast, and bone remodeling.

## miRNA, Osteogenesis, and Bone Formation

Osteoblasts arising from mesenchymal stem cells (MSCs) are responsible for bone matrix synthesis and mineralization during skeletal development and lifelong bone remodeling. The osteogenic differentiation of MSCs and osteoblast‐mediated bone formation are not only governed via the master transcription factors, eg, Runt‐related transcription factor 2 (Runx2) and Osterix, and their downstream signaling cascades, eg, TGF‐β/BMP and Wnt/β‐catenin signaling pathways, but also posttranscriptionally modulated by various miRNAs. The miRNA‐mediated regulatory mechanisms of osteoblast differentiation/functions are summarized in Table 1.

**Table 1:** Selected miRNAs With Their Targets and Functions in Bone Remodeling

miRNA(s)	Target gene(s)	Models/site of action	Function	Reference
miRNA(s) and osteoblasts				
miR‐203	Dlx5	BMP‐2–stimulated human osteoblasts	BMP‐2–stimulated human osteoblast differentiation ↓	Laxman and colleagues^(23)^
miR‐320b				
miR‐214	ATF4	MC3T3‐E1 cells; bone tissues from aged osteoporotic fracture patients; OVX and hindlimb‐unloaded mice	Osteoblast activity and bone formation ↓	Wang and colleagues^(27)^
	Osx	C2C12 cells	Osteogenic differentiation ↓	Shi and colleagues^(29)^
miR‐29a	DKK1, Kremen2, sFRP2	Human osteoblast precursor cell line hFOB1.19; primary cultures of human osteoblasts	Osteogenic differentiation ↑	Kapinas and colleagues^(30)^
miR‐355‐5p	DKK1	HG‐induced apoptosis of MC3T3‐E1 osteoblasts	Activate Wnt signaling	Li and colleagues^(32)^
			Osteogenic differentiation ↑	
miR‐433‐3p	DKK1	Human osteoblast precursor cell line hFOB1.19; primary osteoblasts from ovariectomized rat	Osteoblast differentiation ↑	Tang and colleagues^(33)^
miR‐375‐3p	LRP5, β‐catenin	MC3T3‐E1 osteoblasts	Osteogenesis ↑	Sun and colleagues^(34)^
			Cell apoptosis ↓	
miR‐135	Smad5	BMP‐2–induced C2C12 cells	BMP‐2–induced osteogenic differentiation ↓	Li and colleagues^(35)^
miR‐106b‐5p	Smad5	C2C12 and MC3T3‐E1 cells; OVX mice	Osteogenic differentiation ↓	Fang and colleagues^(36)^
miR‐17‐5p				
miRNA (s) and osteoclasts				
miR‐21	PDCD4	DGCR8 and Dicer knockout BMMs	RANKL‐induced osteoclastogenesis ↑	Sugatani and colleagues^(14)^
	FasL	Primary mouse BMMs	Osteoclastic apoptosis ↓	Sugatani and Hruska^(42)^
miR‐503	RANK	Human CD14^+^ PBMCs; OVX murine model	RANKL‐induced osteoclastogenesis ↓	Chen and colleagues^(49)^
miR‐214‐3p	TRAF3	RAW 264.7 cells; bone specimens from breast cancer patients with osteolytic bone metastasis; human breast cancer–bearing mice; osteoclast‐specific miR‐214‐3p knockout nude mice; osteoclast‐specific miR‐214‐3p knock‐in mice	Osteoclast function in the development of breast cancer osteolytic metastasis	Liu and colleagues^(51)^
	PTEN	RAW 264.7 cells; primary mouse BMMs; osteoclast‐specific miR‐214 transgenic mice	Osteoclast activity ↑	Zhao and colleagues^(52)^
miR‐34a	Tgif2	Primary mouse BMMs; human peripheral blood mononuclear cells; RAW264.7 cells; miR‐34a knockout mice; osteoclastic miR‐34a transgenic mice; osteoclastic miR‐34a conditional knockout mice	Osteoclast differentiation ↓	Krzeszinski and colleagues^(53)^
			Cancer bone metastasis ↓	
miR‐182	Foxo3 and Maml1	Primary mouse BMMs; BMMs from Rbpj^flox/flox^LysMcre(+) mouse	TNF‐α–induced osteoclastogenesis ↑	Miller and colleagues^(56)^
miRNA(s) and osteocytes				
miR‐27a	Prdm16	MC3T3‐E1 cells; Col1a1‐miR‐27a decoy transgenic mice	Osteocyte differentiation ↑	Zeng and colleagues^(58)^
			Enhance TGF‐β signaling to accelerate SOST expression	
miR‐21	PTEN	Cx43‐silenced MLO‐Y4 osteocytic cells, miR21^fl/fl^ mice treated with adenovirus‐Cre	Cx43 maintains osteocyte viability by downstream regulation of miR21 to reduce osteocyte apoptosis	Davis and colleagues^(59)^
miR‐199a‐3p	IGF‐1 and mTOR	MLO‐Y4 osteocytic cells, OVX mice	Osteocytic areas of OVX mice ↑	Fu and colleagues^(60)^
			Estrogen deficiency increases the expression of miR‐199a‐3p to induce autophagy in osteocytes	
miRNA and osteoblast‐osteoclast crosstalk				
miR‐433‐3p (from osteoblasts)	DKK1 (in osteoclasts)	Human osteoblast precursor cell line hFOB1.19; rat ROS17/2.8 cell line; primary rat MSCs; OVX rat model	Relieve the inhibitory effect of DKK1 on osteoblast function	Tang and colleagues^(33)^
miR‐214‐3p (from osteoclasts)	ATF4 (in osteoblasts)	RAW 264.7 cells; OVX mouse; osteoclast‐specific miR‐214‐3p knockout mice; osteoclast‐specific miR‐214‐3p overexpression mice	Osteoblast activity and bone formation ↓	Li and colleagues^(65)^
miR‐218 (from osteocytes)	DKK2 and sFRP2 (in osteoblasts)	Ocy454 osteocytic cells; IDG‐SW3 cells; MC3T3‐E1 cells	Myostatin suppresses osteocyte‐derived exosomal miR‐218 to inhibit osteoblastic differentiation	Qin and colleagues^(69)^

OVX = ovariectomized; HG = high glucose.

### miRNA biogenesis and osteoblast differentiation

Mice with *Dicer* deletion in *Prx1+* mesenchymal osteochondroprogenitor cells (*Prx1‐Cre*;*Dicer* ^*flox/flox*^) were viable, but exhibited significant skeletal defects including reduced hindlimb size and twisted bone.^15^ Interestingly, in later research, Gaur and colleagues^11^ reported that conditional excision of the Dicer enzyme in *Col1α1*+ osteoblast lineage cells (*Col1α1‐Cre*;*Dicer* ^*flox/flox*^) is deleterious to fetal survival. Impressively, the embryonic day 14.5 (E14.5) Dicer‐mutant fetal pups showed a deformed cartilaginous skeleton with impaired bone formation. Both studies suggest that the Dicer‐mediated miRNA processing mechanism is required for the proper hindlimb morphogenesis and skeletal development, whereas the differences in fetal survival between the *Prx1‐Cre*;*Dicer* ^*flox/flox*^ and *Col1α1‐Cre*;*Dicer* ^*flox/flox*^ mice could be due to the different *Dicer* ^*flox/flox*^ mouse strain used. Conversely, a recent study showed that mice with DGCR8 conditional deletion in *Col1α1*+ osteoblast lineage cells (*Col1α1‐Cre*;*DGCR8* ^*flox/flox*^) exhibited increased osteoblastic bone formation,^10^ suggesting that the DROSHA/DGCR8‐mediated miRNA processing pathway could negatively regulate osteoblast activity and bone formation in a Dicer‐independent manner.

On the other hand, to overcome the detrimental effect of *Dicer* inactivation on fetal survival, Bendre and colleagues^9^ generated an inducible pre‐osteoblast specific Dicer1 knockout model by employing tamoxifen‐controllable Cre allele (*Sp7‐Cre*/ERT2;*Dicer* ^*flox/flox*^). They found that tamoxifen‐dependent inactivation of Dicer1 in osterix+ preosteoblasts dramatically impaired the bone formation of cortical bone but not trabecular bone in both prepubertal and adult mice, suggesting an important role of Dicer‐processed miRNAs in the postnatal regulation of cortical bone homeostasis. Consistently, Liu and colleagues^16^ showed that ablation of *Dicer* in Runx2+ osteoblast lineage cells (*Runx2‐Cre*;*Dicer* ^*flox/flox*^) did not induce embryonic lethality, although it could cause remarkable growth retardation, low bone density, and impaired bone formation during postnatal development. Interestingly, they did not find significant difference in the glucocorticoid‐induced bone formation reduction between the *Runx2‐Cre*;*Dicer* ^*flox/flox*^ mice and littermate control mice upon glucocorticoid (GC) treatment. In addition, Gaur and colleagues^11^ found that the mice with *Dicer* deletion in osteocalcin‐expressing mature osteoblasts (*Ocn‐Cre*; *Dicer* ^*flox/flox*^) were also viable with a perinatal phenotype of delayed bone mineralization, which returned to normal at 1 month of age. Surprisingly, they further observed a second phenotype of significantly increased bone mass developed by 2 months, which continued up to 8 months in long bones and vertebrae.^11^ Collectively, these findings indicate that the Dicer‐processed miRNAs in early osteoprogenitors are essential for osteogenesis and bone formation, whereas loss of the Dicer‐processed miRNAs in mature osteoblasts seem to have anabolic effect on the adult skeleton.

In turn, the miRNA expression and Dicer‐mediated miRNA processing mechanism were under control by the osteogenic transcription factor during osteoblast lineage commitment. Zhou and colleagues^12^ observed the coincident expression of Dicer and Runx2 during osteogenesis differentiation of mouse MC3T3‐E1 preosteoblasts. They further witnessed that Runx2 could directly bind to the Dicer promoter region to enhance Dicer expression.^12^ In addition, by comparing the miRNA expression in calvaria of the E18.5 *Osx* gene knockout embryos with wild‐type embryos and verifying in osteoblasts overexpressing *Osx*, Chen and colleagues^17^ identified a group of miRNAs that was downregulated by *Osx* expression, including miR‐133a, miR‐204, miR‐211, miR‐302a, miR‐433, miR‐501, and miR‐544. They also found another group of miRNAs that was upregulated by *Osx* expression, including miR‐141, miR‐200a, miR‐192, and miR‐1194.^17^

### Osteoblastic miRNA and osteogenic transcription factor

Runx2 is the master transcription factor for osteoblast differentiation. In a study by Zhang and colleagues,^18^ they found that a panel of 11 Runx2‐targeting miRNAs (miR‐23a, miR‐30c, miR‐34c, miR‐133a, miR‐135a, miR‐137, miR‐204, miR‐205, miR‐217, miR‐218, and miR‐338) were inversely expressed relative to Runx2 during osteogenic differentiation of mouse MC3T3 E1 osteoblastic cells and chondrogenic differentiation of mouse ATDC5 prechondrocytes. Specifically, the expression of these miRNAs was remarkably upregulated at a late stage of osteoblast maturation when Runx2 protein expression was decreased and downregulated at late stage of hypertrophic chondrocyte differentiation. They further demonstrated that all these miRNAs could directly target and downregulate the Runx2 protein expression. These results corroborate the previous study mentioned above showing that excision of the miRNA processing enzyme Dicer in mature osteoblasts causes a dramatic high bone mass phenotype,^11^ indicating that the Runx2‐targeting miRNAs are generally required for attenuating osteoblast maturation. In addition, several independent studies have reported that Runx2 could be directly regulated by other miRNAs, such as miR‐30d,^19^ miR‐467g,^20^ and miR‐628‐3p.^21^

The homeodomain protein Distal‐less Homeobox 5 (Dlx5) is an essential activator of Runx2 and Osterix (Osx).^22^ A study by Laxman and colleagues^23^ reported that miR‐203 and miR‐320b could negatively regulate BMP‐2‐stimulated human osteoblast differentiation by inhibiting Dlx5, which in turn suppresses the downstream osteogenic master transcription factor Runx2 and Osx to hamper osteoblast differentiation. The activating transcription factor 4 (ATF4) is another bone‐related transcription factor critical for the proliferation, differentiation, and survival of osteoblasts.^24^, ^25^, ^26^ Our laboratory has shown that miR‐214 could directly target ATF4 to inhibit osteoblast activity and bone formation.^27^ We identified that miR‐214‐3p, among the most highly expressed miRNAs within bone tissues from aged osteoporotic fracture patients, could downregulate the amount of ATF4 proteins in osteoblasts to contribute to both age‐related and hindlimb unloading–induced bone formation reduction. In addition, miR‐214 was also reported to posttranscriptionally regulate the expression of Osx, another master transcription factor for osteoblast differentiation expression.^28^ Shi and colleagues^29^ found that miR‐214 could directly target two binding site of Osx 3′UTR to inhibit the Osx protein expression for suppressing the osteogenic differentiation of C2C12 cells.

### Osteoblastic miRNA and osteogenic signal

The two crucial osteogenic signals, ie, the Wnt/β‐catenin and BMP signaling pathway, are regulated by miRNAs. A previous study found that the negative regulators of Wnt signaling, including Dikkopf‐1 (DKK1), Kremen2, and secreted frizzled related protein 2 (sFRP2), were the direct targets of miR‐29a.^30^ The expression of miR‐29a was increased during osteogenic differentiation in the human osteoblast precursor cell line hFOB1.19 as well as in primary cultures of human osteoblasts. Transfection with miR‐29a inhibitor increased the endogenous protein levels of the aforementioned Wnt antagonists, whereas transfection with miR‐29a mimics decreased the endogenous protein levels of the aforementioned Wnt antagonists, and therefore, suppressed and potentiated the Wnt signaling.^30^ In another study, Zhang and colleagues^31^ showed that miR‐335‐5p could activate Wnt signaling and promote osteogenic differentiation *via* directly targeting and downregulating DKK1. Consistently, Li and colleagues^32^ found that overexpression of miR‐335‐5p could decrease the protein expression levels of DKK1to inhibit the high‐glucose (HG)‐induced apoptosis of MC3T3‐E1 osteoblasts. In addition, Tang and colleagues^33^ observed a positive correlation between the serum DKK1 levels and circulating miR‐433‐3p levels in ovariectomized (OVX) rats, and further showed that miR‐433‐3p could target DKK1 to promote osteoblast differentiation in vitro. On the other hand, Sun and colleagues^34^ showed that the LRP5, a co‐receptor of the Wnt signaling and β‐catenin, the downstream signal transducer of the Wnt signaling, were both the targets of miR‐375‐3p. They found that transfection of miR‐375‐3p in MC3T3‐E1 osteoblasts not only arrested the protein expression of LRP5 and β‐catenin, but also impaired osteogenesis and induced cell apoptosis.

Li and colleagues^35^ found that the expression of miR‐135 was decreased during BMP‐2–induced osteogenesis of C2C12 cells. They further showed that miR‐135 could directly target Smad5, a key transducer of the osteogenic BMP signal, to inhibit the BMP‐2–induced osteogenic differentiation. Consistently, Fang and colleagues^36^ identified that miR‐106b‐5p and miR‐17‐5p could both suppress the osteogenic differentiation of C2C12 and MC3T3‐E1 cells by targeting Smad5. Inhibition of miR‐106b‐5p and miR‐17‐5p in OVX mice could result in increased bone formation as well as improvement of trabecular microarchitecture.

## miRNA, Osteoclastogenesis, and Bone Resorption

Osteoclasts derived from bone marrow monocyte‐macrophage (BMM) precursors are the primary bone‐resorbing cells. Osteoclastogenesis involving the fusion of precursors to form multinucleated osteoclasts is regulated by two essential cytokines; ie, macrophage colony‐stimulating factor‐1 (M‐CSF) and receptor activator of NFκB ligand (RANKL). An increasing line of evidence suggests that miRNAs also play critical roles in regulating osteoclastogenesis and bone resorption. The miRNA‐mediated regulatory mechanisms of osteoclast differentiation/functions are summarized in Table 1.

### miRNA biogenesis and osteoclast differentiation

Sugatani and Hruska^37^ found that the RANKL‐induced expression of osteoclastic transcription factors and their function in osteoclast precursors were inhibited, together with the osteoclastogenesis and bone resorption by small interfering RNA‐mediated silencing of either DGCR8, Dicer, or Ago2. By genetic approach, their CD11b‐Cre/Dicer^fl/fl^ mice lacking Dicer in CD11b+ osteoclast precursors exhibited a mild osteopetrosis phenotype caused by decreased osteoclast formation and impaired bone resorption. Consistently, another study by Mizoguchi and colleagues^13^ showed that depletion of Dicer gene in Cathepsin (Ctsk)‐expressing osteoclasts at a more mature stage also caused decreased osteoclast formation and bone resorption in vivo, as well as impaired osteoclastic activity in vitro. In line with the bone phenotype in the aforementioned Dicer mutant mice,^13^, ^37^ Sugatani and colleagues^38^ found that osteoclast‐specific deletion of DGCR8 (Ctsk‐Cre/DGCR8^fl/fl^) resulted in impaired osteoclastic development and bone resorption. Taken together, both the DGCR8‐dependent miRNA biogenesis and Dicer‐dependent miRNA processing are indispensable for osteoclastogenesis and osteoclastic bone resorption.

### Osteoclastic miRNA and osteoclastogenesis

Apart from its critical role in tumor growth and invasion,^39^, ^40^, ^41^ miR‐21 is one of the most commonly studied pro‐osteoclastogenic miRNAs identified so far. Sugatani and colleagues^14^ had profiled the miRNA expression in RANKL‐induced BMM osteoclastogenesis and identified that miR‐21, among the 38 upregulated miRNAs, was robustly stimulated by RANKL. They documented that RANKL induced the expression of c‐Fos that stimulates miR‐21 expression, whereas miR‐21 could directly target and downregulate the programmed cell death 4 (PDCD4) to remove the repression from c‐Fos. Consistently, they found that BMMs deficient in either the DGCR8 or Dicer gene possessed significantly decreased miR‐21 levels and increased PDCD4 protein levels, but impaired capacity for RANKL‐induced osteoclastogenesis. Interestingly, they showed that forced expression of miR‐21 could downregulate the PDCD4 protein expression to rescue the osteoclast development in both DGCR8 and Dicer knockout BMMs. However, it remains elusive whether such a rescue effect of miR‐21 overexpression was Dicer‐independent in Dicer‐deficient BMMs. The same research team reported in a later study that estrogen could enhance the protein expression of FasL, the target of miR‐21, through downregulating miR‐21 biogenesis, and therefore induce osteoclastic apoptosis.^42^ The pro‐osteoclastogenic effect of miR‐21 was also confirmed in the miR‐21 global knockout mice in vivo.^43^

The miR‐29 family members, including miR‐29a/b/c, were reported to regulate murine osteoclast commitment and migration.^44^ The study by Franceschetti and colleagues^44^ found that the expression of miR‐29a/b/c increased during osteoclast differentiation, in concert with mRNAs for the osteoclast markers Acp5 and Ctsk. Intriguingly, by inducible miR‐29 inhibition, they found that miR‐29 knockdown hampered the migration and osteoclastic commitment of preosteoclasts without affecting the cell viability, actin ring formation, or apoptosis in mature osteoclasts. Their luciferase reporter assay validated that miR‐29 could directly target the mRNAs of cytoskeletal organization‐associated molecules, including cell division control protein 42 (Cdc42) and SLIT‐ROBO Rho GTPase‐activating protein 2 (Srgap2), the mRNAs of macrophage lineage‐associated proteins, including G protein‐coupled receptor 85 (Gpr85), nuclear factor I/A (Nfia), and Cd93, and the mRNA of calcitonin receptor (Ctr) that regulates osteoclast survival and resorption. However, in another in vivo study by Wang and colleagues,^45^ they found that gain of miR‐29a function in rats by administering lentivirus‐mediated miR‐29a precursor not only alleviated the detrimental effects of glucocorticoid treatment on mineral acquisition and ex vivo osteoblast differentiation, but also reduced osteoclast surface, ex vivo osteoclast differentiation, and RANKL expression in bone microenvironments. In turn, miR‐29 knockdown in rats by administering lentivirus‐mediated miR‐29a inhibitor accelerated osteoclast resorption, cortical bone porosity and fragility, as well as the loss of ex vivo osteogenic differentiation capacity. Because the mature miR‐29a/b/c are highly conserved in human, mouse, and rat,^46^ these controversial outcomes between manipulating miR‐29a and miR‐29 family on osteoclast activity may indicate the independent role of each miR‐29 family members in regulating osteoclast.

### Osteoclastic miRNA and osteoporosis

Li and colleagues^47^ detected the upregulated miR‐133a in serum isolated from postmenopausal osteoporosis patients, which was negatively correlated with the patients’ lumbar bone mineral density (BMD). They demonstrated that miR‐133a knockdown could inhibit the RANKL‐induced osteoclastogenesis in vitro and alleviated the bone loss in ovariectomized rats in vivo. Cheng and colleagues^48^ identified that miR‐148a, among the most upregulated miRNAs during the M‐CSF and RANKL‐stimulated osteoclast differentiation of human circulating CD14+ peripheral blood mononuclear cells (PBMCs), could directly target and downregulate the V‐maf musculoaponeurotic fibrosarcoma oncogene homolog B (MAFB) to promote osteoclastogenesis. They further showed that CD14+ PBMCs from lupus patients possessed elevated miR‐148a levels and enhanced osteoclastogenesis capacity, which may contribute to the lower BMD in lupus patients compared with normal controls. In another study by the same research team, Chen and colleagues^49^ showed that miR‐503 was markedly downregulated in circulating CD14+ PBMC from postmenopausal osteoporosis patients compared with those from postmenopausal healthy women. Mechanistically, they verified that miR‐503 could directly target RANK to dampen the RANKL‐induced osteoclastogenesis. These findings from patients with osteoporosis would provide new miRNA‐based disease biomarkers and therapeutic targets for developing novel anti resorption treatment.

### Osteoclastic miRNA and osteolytic bone metastasis

Ell and colleagues^50^ profiled the miRNA expression in osteoclast differentiation induced by conditioned media from highly metastatic breast cancer cells. They identified a series of tumor‐suppressed miRNAs, including miR‐33a, miR‐133a, miR‐141, miR‐190, and miR‐219, that exert inhibitory effect on tumor‐induced osteoclastogenesis, and two tumor‐induced miRNAs, ie, miR‐378 and miR‐16, that are elevated during tumor‐induced osteoclastogenesis and correlate with bone metastasis burden. The study has provided experimental and clinical evidence to delineate the role of miRNAs in regulating osteolytic bone metastasis. It is interesting to note that miR‐133a was found to inhibit osteoclast differentiation and resorption activity in vitro, in contrast to the aforementioned positive regulatory role of miR‐133a in osteoclasts,^47^ which may attribute to the different disease mechanism between osteoporosis and cancer bone metastasis. In addition, our laboratory has identified that miR‐214‐3p was significantly upregulated in bone specimens from breast cancer patients with osteolytic bone metastasis.^51^ We showed that miR‐214‐3p could directly regulate the protein expression TRAF3 rather than phosphatase and tensin homolog (PTEN),^24^ the previously verified miR‐214‐3p target in osteoclasts,^52^ to promote osteoclast function in the development of breast cancer osteolytic metastasis. Moreover, the study by Krzeszinski and colleagues^53^ reported an inhibitory role of miR‐34a on osteoclast differentiation and cancer bone metastasis. They identified transforming growth factor‐b‐induced factor 2 (Tgif2) as a direct target of miR‐34a. They proved that ovariectomy‐induced osteoporosis, as well as bone metastasis of breast and skin cancers, is almost prevented in osteoclastic miR‐34a transgenic mice and can be effectively attenuated by miR‐34a nanoparticle treatment.

### Osteoclastic miRNA and inflammatory response

Tumor necrosis factor alpha (TNF‐α), a proinflammatory cytokine involved in the pathogenesis of chronic inflammatory diseases, could stimulates osteoclast differentiation in a Rankl–Rank independent mechanism.^54^ In a previous study with microarray screening, it was found that miR‐378, miR‐21, miR‐29b, miR‐146a, miR‐155, and miR‐210 were highly expressed, while miR‐223 was downregulated during TNF‐α–induced osteoclast differentiation of murine BMMs. The expression profile of osteoclast miRNAs with TNF‐α stimulation was partly matched with the previous profile outcomes of pro‐osteoclastogenic miRNA without TNF‐α stimulation.^38^, ^44^, ^50^ The transcription factor RBP‐J is a newly identified osteoclastogenic repressor playing a critical role in inhibiting the TNF‐α–induced osteoclast differentiation and bone resorption.^55^ Miller and colleagues^56^ recently found that miR‐182, as the most abundant miRNA in TNF‐α–induced osteoclastogenesis, was repressed by RBP‐J during osteoclast differentiation. miR‐182 could promote the TNF‐α–induced osteoclastogenesis via inhibition of Foxo3 and Maml1. Therefore, it proposes an important mechanism by which suppression of miR‐182 by RBP‐J may restrain TNF‐α–induced osteoclastogenesis.

## miRNA and Osteocytes

Osteocytes are the terminally differentiated cell type of the osteoblastic lineage, accounting for ~98% of the cells comprising the skeleton. They are mechanosensitive cells embedded in the bone matrix that have crucial functions in regulating skeletal homeostasis.^57^ However, unlike osteoblasts and osteoclasts, the potential role of miRNA‐mediated regulation in osteocytes is just starting to be uncovered. The miRNA‐mediated regulatory mechanisms of osteocyte differentiation/functions are summarized in Table 1.

Eguchi and colleagues^19^ performed RT‐qPCR microarray analysis to examine the miRNA expression profiling in osteocytogenesis of murine bone‐marrow–derived mesenchymal stem cell line KUSA‐A1, by which they identified the upregulated miRNAs, including miR‐30d, miR‐155, miR‐21, miR‐16, miR‐34c, miR‐18ab, miR‐19, miR‐541, and miR‐23a, and the downregulated miRNAs including let‐7/miR98, during osteocytic differentiation. Interestingly, miR30d, miR‐155, miR‐21, miR‐34c, and miR‐16, among the upregulated miRNAs were all predicted to repress mRNAs of osteoblast stemness‐related genes or key osteoblastic factors including several key osteoblastic factors RUNX2, NOTCH1, SMAD1/2/4/7, SOX2/9, TGFBR2, BMPR1A, and LRP6 and CCN3. In addition, miR‐18ab and miR‐19 were predicted to target the osteochondrogenesis factors CTGF/CCN2. On the other hand, the downregulated miRNAs, eg, let‐7/miR‐98, were predicted to target and repress mRNA expression of osteocyte‐specific dentin matrix protein 1 (DMP1). Consistently, another study conducted by Zeng and colleagues^58^ showed that the miR‐23a cluster, containing miR23a, miR27a and miR24‐2, could promote osteocyte differentiation. By genetic approach, they found that the osteoblast‐specific miR‐23a cluster gain‐of‐function mice exhibited low bone mass associated with decreased osteoblast but increased osteocyte numbers, whereas the loss‐of‐function transgenic mice overexpressing miRNA decoys for either miR‐23a or miR‐27a showed decreased osteocyte numbers. Moreover, they identified that the upregulated miR‐23a cluster could directly target and repress Prdm16 for enhancing the TGF‐β signaling to accelerate the expression of sclerostin during osteocytic differentiation. In line with the above microarray data, Davis and colleagues^59^ found that the miR‐21 expression was markedly downregulated in connexin43 (Cx43)‐silenced MLO‐Y4 osteocytic cells that undergo spontaneous cell death in culture. Similarly, the bones from Cx43‐deficient mice and 24‐month‐old mice both exhibit reduced levels of the miR‐21 and increased levels of the miR‐21 target PTEN. They further demonstrated that miR‐21 lies downstream of Cx43 to repress PTEN for reducing osteocyte apoptosis. In addition, Fu and colleagues^60^ found that miR‐199a‐3p could mediate the osteocyte autophagy. They observed that miR‐199a‐3p expression was upregulated in osteocytic areas of OVX mice with estrogen deficiency. Mechanistically, a series of their in vitro data from MLO‐Y4 cells documented that estrogen deficiency increased the expression of miR‐199a‐3p, which could induce autophagy in osteocytes via targeting insulin growth factor‐1 (IGF‐1) and mammalian target of rapamycin (mTOR) to repress the mTOR‐related signaling cascades.

## miRNA and Bone Cell Crosstalk

Besides their intracellular function, emerging studies have uncovered that miRNAs can traffic in exosomes serving as intercellular signals to mediate cell‐cell communications.^61^, ^62^

Evidence of the exosomal miRNA‐mediated crosstalk is increasingly witnessed in bone cells and is being extensively investigated.^63^, ^64^ Our laboratory has identified that exosomal miR‐214‐3p secreted by osteoclasts was transferred to osteoblasts to inhibit osteoblast activity and bone formation.^65^ Consistently, another study further demonstrated that ephrinA2 and EphA2 interaction could facilitate the recognition of osteoclast‐derived exosome by osteoblasts.^66^ On the other hand, a recent study also postulated that the miR‐433‐3p highly expressed by osteoblasts could be secreted in osteoblast‐derived exosomes for targeting DKK1 expression in osteoclasts, which in turn relieves the inhibitory effect of DKK1 on osteoblast function.^33^ In another study, Cui and colleagues^67^ found that MC3T3 mouse osteoblasts could release exosomes containing osteogenic miRNAs to promote the osteoblast differentiation of the recipient ST2 cells. In addition, a recent study showed that ablation of osteocytes in a transgenic (DMP‐1 DTR Tg) mouse with targeted expression of diphtheria toxin receptor (DTR) under the promoter of DMP‐1 resulted in the downregulated expression of 12 miRNAs (miR‐3473a, miR‐3473b, miR‐3473e, miR‐5128, miR‐6244, miR‐6239, miR‐5132, miR‐705, miR‐208a, miR‐3104, miR‐1224, and miR‐5621) in serum exosomes,^68^ suggesting that osteocyte could also release miRNA‐containing exosomes for cell‐cell communication. Interestingly, Qin and colleagues^69^ found that Myostatin, a myokine secreted by muscles, could suppress miR‐218 expression in Ocy454 osteocytes and their exosomes. The Myostatin‐treated Ocy454 cell‐derived exosomes could inhibit the osteoblastic differentiation of MC3T3 cells, which could be reversed by introduction of miR‐218 mimics in Ocy454 exosomes. With the rising interest in this area, it would be so exciting to establish the physiological/pathological role of miRNA‐mediated crosstalk among bone cells as well as between bone and other organs, and thereafter, develop new therapeutic agents targeting the adverse crosstalk in bone diseases. The miRNA‐mediated mechanisms in bone cell crosstalk are summarized in Table 1.

## Summary and Prospective

**Figure 1:**
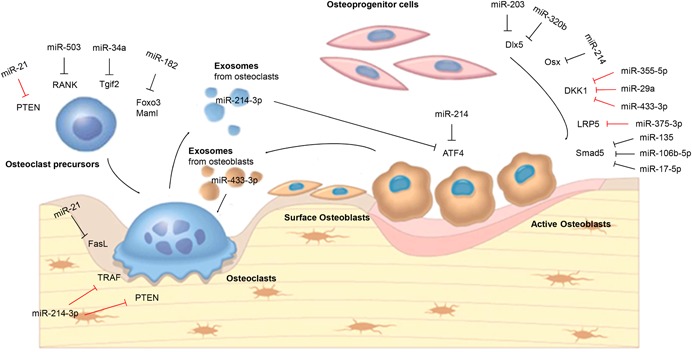
Schematic diagram of the key miRNA players in osteoblast differentiation, osteoclast differentiation, and osteoblast‐osteoclast crosstalk. Red lines ending with a short perpendicular line indicate that miRNA‐mediated regulation upregulates the osteoblast/osteoclast differentiation and activity. Black lines ending with a short perpendicular line indicate that miRNA‐mediated regulation downregulates the osteoblast/osteoclast differentiation and activity.

In summary, miRNA‐mediated posttranscriptional regulation is a highly efficient regulatory mechanism for orchestrating the physiological activity of osteoblasts, osteoclasts, and osteocytes (Fig. 1).^70^, ^71^ However, the dysregulation of miRNAs always results in impaired osteoblast, osteoclast, and osteocyte function, leading to abnormal bone remodeling. In addition, miRNA‐mediated crosstalk not only represents a novel paracrine‐like mechanism for coupling osteoblast and osteoclast function, but also may contribute to pathological uncoupling of bone formation and bone resorption. More in‐depth studies are still required to uncover the upstream molecular events conducting the miRNA expression and to build up a miRNA‐regulatory network in specific bone cells.

## Disclosures

All authors state that they have no conflicts of interest.

## References

[1] Zaidi M . Skeletal remodeling in health and disease. Nat Med. 2007;13:791–801. 17618270 10.1038/nm1593

[2] Reppe S , Datta HK , Gautvik KM . Omics analysis of human bone to identify genes and molecular networks regulating skeletal remodeling in health and disease. Bone. 2017;101:88–95. 28450214 10.1016/j.bone.2017.04.012

[3] Bartel DP . MicroRNAs: genomics, biogenesis, mechanism, and function. Cell. 2004;116:281–97. 14744438 10.1016/s0092-8674(04)00045-5

[4] Bernstein E , Caudy AA , Hammond SM , Hannon GJ . Role for a bidentate ribonuclease in the initiation step of RNA interference. Nature. 2001;409:363–6. 11201747 10.1038/35053110

[5] Gregory RI , Yan KP , Amuthan G , et al. The Microprocessor complex mediates the genesis of microRNAs. Nature. 2004;432(7014):235–40. 15531877 10.1038/nature03120

[6] Denli AM , Tops BB , Plasterk RH , Ketting RF , Hannon GJ . Processing of primary microRNAs by the Microprocessor complex. Nature. 2004;432(7014):231–5. 15531879 10.1038/nature03049

[7] Kwon SC , Nguyen TA , Choi YG , et al. Structure of human DROSHA. Cell. 2016;164:81–90. 26748718 10.1016/j.cell.2015.12.019

[8] Wu Q , Song R , Ortogero N , et al. The RNase III enzyme DROSHA is essential for microRNA production and spermatogenesis. J Biol Chem 2012;287:25173–90. 22665486 10.1074/jbc.M112.362053 PMC3408133

[9] Bendre A , Moritz N , Vaananen V , Maatta JA . Dicer1 ablation in osterix positive bone forming cells affects cortical bone homeostasis. Bone. 2018;106:139–47. 29066312 10.1016/j.bone.2017.10.018

[10] Choi YJ , Jeong S , Yoon KA , et al. Deficiency of DGCR8 increases bone formation through downregulation of miR‐22 expression. Bone. 2017;103:287–94. 28739418 10.1016/j.bone.2017.07.021

[11] Gaur T , Hussain S , Mudhasani R , et al. Dicer inactivation in osteoprogenitor cells compromises fetal survival and bone formation, while excision in differentiated osteoblasts increases bone mass in the adult mouse. Dev Biol. 2010;340(1):10–21. 20079730 10.1016/j.ydbio.2010.01.008 PMC2840721

[12] Zhou J , Hu Y , Chen Y , et al. Dicer‐dependent pathway contribute to the osteogenesis mediated by regulation of Runx2. Am J Transl Res 2016;8:5354–69. 28078008 PMC5209488

[13] Mizoguchi F , Izu Y , Hayata T , et al. Osteoclast‐specific Dicer gene deficiency suppresses osteoclastic bone resorption. J Cell Biochem 2010;109:866–75. 20039311 10.1002/jcb.22228

[14] Sugatani T , Vacher J , Hruska KA . A microRNA expression signature of osteoclastogenesis. Blood. 2011;117:3648–57. 21273303 10.1182/blood-2010-10-311415 PMC3072882

[15] Harfe BD , McManus MT , Mansfield JH , Hornstein E , Tabin CJ . The RNaseIII enzyme Dicer is required for morphogenesis but not patterning of the vertebrate limb. Proc Natl Acad Sci U S A 2005;102:10898–903. 16040801 10.1073/pnas.0504834102 PMC1182454

[16] Liu P , Baumgart M , Groth M , et al. Dicer ablation in osteoblasts by Runx2 driven cre‐loxP recombination affects bone integrity, but not glucocorticoid‐induced suppression of bone formation. Sci Rep. 2016;6:32112. 27554624 10.1038/srep32112 PMC4995469

[17] Chen Q , Liu W , Sinha KM , Yasuda H , de Crombrugghe B . Identification and characterization of microRNAs controlled by the osteoblast‐specific transcription factor Osterix. PLoS One. 2013;8:e58104. 23472141 10.1371/journal.pone.0058104 PMC3589352

[18] Zhang Y , Xie RL , Croce CM , et al. A program of microRNAs controls osteogenic lineage progression by targeting transcription factor Runx2. Proc Natl Acad Sci U S A 2011;108:9863–8. 21628588 10.1073/pnas.1018493108 PMC3116419

[19] Eguchi T , Watanabe K , Hara ES , Ono M , Kuboki T , Calderwood SK . OstemiR: a novel panel of microRNA biomarkers in osteoblastic and osteocytic differentiation from mesenchymal stem cells. PLoS One. 2013;8:e58796. 23533592 10.1371/journal.pone.0058796 PMC3606401

[20] Kureel J , John AA , Dixit M , Singh D . MicroRNA‐467g inhibits new bone regeneration by targeting Ihh/Runx‐2 signaling. Int J Biochem Cell Biol 2017;85:35–43. 28163186 10.1016/j.biocel.2017.01.018

[21] Chen H , Ji X , She F , Gao Y , Tang P . miR‐628‐3p regulates osteoblast differentiation by targeting RUNX2: possible role in atrophic non‐union. Int J Mol Med 2017;39:279–86. 28035362 10.3892/ijmm.2016.2839 PMC5358698

[22] Miyama K , Yamada G , Yamamoto TS , et al. A BMP‐inducible gene, dlx5, regulates osteoblast differentiation and mesoderm induction. Dev Biol. 1999;208:123–33. 10075846 10.1006/dbio.1998.9197

[23] Laxman N , Mallmin H , Nilsson O , Kindmark A . miR‐203 and miR‐320 regulate bone morphogenetic protein‐2‐induced osteoblast differentiation by targeting distal‐less homeobox 5 (Dlx5). Genes (Basel) 2016;8:4 DOI: 10.3390/genes8010004 PMC5294999 28025541

[24] Yang X , Matsuda K , Bialek P , et al. ATF4 is a substrate of RSK2 and an essential regulator of osteoblast biology; implication for Coffin‐Lowry Syndrome. Cell. 2004;117:387–98. 15109498 10.1016/s0092-8674(04)00344-7

[25] Yang X , Karsenty G . ATF4, the osteoblast accumulation of which is determined post‐translationally, can induce osteoblast‐specific gene expression in non‐osteoblastic cells. J Biol Chem 2004;279:47109–14. 15377660 10.1074/jbc.M410010200

[26] Zhang X , Yu S , Galson DL , et al. Activating transcription factor 4 is critical for proliferation and survival in primary bone marrow stromal cells and calvarial osteoblasts. J Cell Biochem 2008;105:885–95. 18729081 10.1002/jcb.21888 PMC2704124

[27] Wang X , Guo B , Li Q , et al. miR‐214 targets ATF4 to inhibit bone formation. Nat Med. 2013;19:93–100. 23223004 10.1038/nm.3026

[28] Nakashima K , Zhou X , Kunkel G , et al. The novel zinc finger‐containing transcription factor osterix is required for osteoblast differentiation and bone formation. Cell. 2002;108:17–29. 11792318 10.1016/s0092-8674(01)00622-5

[29] Shi K , Lu J , Zhao Y , et al. MicroRNA‐214 suppresses osteogenic differentiation of C2C12 myoblast cells by targeting Osterix. Bone. 2013;55:487–94. 23579289 10.1016/j.bone.2013.04.002

[30] Kapinas K , Kessler C , Ricks T , Gronowicz G , Delany AM . miR‐29 modulates Wnt signaling in human osteoblasts through a positive feedback loop. J Biol Chem 2010;285:25221–31. 20551325 10.1074/jbc.M110.116137 PMC2919085

[31] Zhang J , Tu Q , Bonewald LF , et al. Effects of miR‐335‐5p in modulating osteogenic differentiation by specifically downregulating Wnt antagonist DKK1. J Bone Miner Res 2011;26:1953–63. DOI: 10.1002/jbmr.377 21351149 PMC3810406

[32] Li J , Feng Z , Chen L , Wang X , Deng H . MicroRNA‐335‐5p inhibits osteoblast apoptosis induced by high glucose. Mol Med Rep 2016;13:4108–12. 26986081 10.3892/mmr.2016.4994

[33] Tang X , Lin J , Wang G , Lu J . MicroRNA‐433‐3p promotes osteoblast differentiation through targeting DKK1 expression. PLoS One. 2017;12:e0179860. 28628652 10.1371/journal.pone.0179860 PMC5476290

[34] Sun T , Li CT , Xiong L , et al. miR‐375‐3p negatively regulates osteogenesis by targeting and decreasing the expression levels of LRP5 and beta‐catenin. PLoS One. 2017;12:e0171281. 28158288 10.1371/journal.pone.0171281 PMC5291413

[35] Li Z , Hassan MQ , Volinia S , et al. A microRNA signature for a BMP2‐induced osteoblast lineage commitment program. Proc Natl Acad Sci U S A 2008;105:13906–11. 18784367 10.1073/pnas.0804438105 PMC2544552

[36] Fang T , Wu Q , Zhou L , Mu S , Fu Q . miR‐106b‐5p and miR‐17‐5p suppress osteogenic differentiation by targeting Smad5 and inhibit bone formation. Exp Cell Res 2016;347:74–82. 27426726 10.1016/j.yexcr.2016.07.010

[37] Sugatani T , Hruska KA . Impaired micro‐RNA pathways diminish osteoclast differentiation and function. J Biol Chem 2009;284:4667–78. 19059913 10.1074/jbc.M805777200 PMC2640963

[38] Sugatani T , Hildreth BE 3rd , Toribio RE , Malluche HH , Hruska KA . Expression of DGCR8‐dependent microRNAs is indispensable for osteoclastic development and bone‐resorbing activity. J Cell Biochem 2014;115:1043–7. DOI: 10.1002/jcb.24759 24420069 PMC4079251

[39] Asangani IA , Rasheed SA , Nikolova DA , et al. MicroRNA‐21 (miR‐21) post‐transcriptionally downregulates tumor suppressor Pdcd4 and stimulates invasion, intravasation and metastasis in colorectal cancer. Oncogene. 2008;27:2128–36. 17968323 10.1038/sj.onc.1210856

[40] Frankel LB , Christoffersen NR , Jacobsen A , Lindow M , Krogh A , Lund AH . Programmed cell death 4 (PDCD4) is an important functional target of the microRNA miR‐21 in breast cancer cells. J Biol Chem 2008;283:1026–33. 17991735 10.1074/jbc.M707224200

[41] Kumarswamy R , Volkmann I , Thum T . Regulation and function of miRNA‐21 in health and disease. RNA Biol. 2011;8:706–13. 21712654 10.4161/rna.8.5.16154 PMC3256347

[42] Sugatani T , Hruska KA . Down‐regulation of miR‐21 biogenesis by estrogen action contributes to osteoclastic apoptosis. J Cell Biochem 2013;114:1217–22. 23238785 10.1002/jcb.24471 PMC4154486

[43] Hu CH , Sui BD , Du FY , et al. miR‐21 deficiency inhibits osteoclast function and prevents bone loss in mice. Sci Rep. 2017;7:43191. 28240263 10.1038/srep43191 PMC5327426

[44] Franceschetti T , Kessler CB , Lee SK , Delany AM . miR‐29 promotes murine osteoclastogenesis by regulating osteoclast commitment and migration. J Biol Chem 2013;288:33347–60. 24085298 10.1074/jbc.M113.484568 PMC3829182

[45] Wang FS , Chuang PC , Lin CL , et al. MicroRNA‐29a protects against glucocorticoid‐induced bone loss and fragility in rats by orchestrating bone acquisition and resorption. Arthritis Rheum 2013;65:1530–40. DOI: 10.1002/art.37948 23529662

[46] Kriegel AJ , Liu Y , Fang Y , Ding X , Liang M . The miR‐29 family: genomics, cell biology, and relevance to renal and cardiovascular injury. Physiol Genomics. 2012;44:237–44. 22214600 10.1152/physiolgenomics.00141.2011 PMC3289120

[47] Li Z , Zhang W , Huang Y . MiRNA‐133a is involved in the regulation of postmenopausal osteoporosis through promoting osteoclast differentiation. Acta Biochim Biophys Sin (Shanghai) 2018;50:273–80. DOI: 10.1093/abbs/gmy006 29425279

[48] Cheng P , Chen C , He HB , et al. miR‐148a regulates osteoclastogenesis by targeting V‐maf musculoaponeurotic fibrosarcoma oncogene homolog B. J Bone Miner Res 2013;28:1180–90. DOI: 10.1002/jbmr.1845 23225151

[49] Chen C , Cheng P , Xie H , et al. MiR‐503 regulates osteoclastogenesis via targeting RANK. J Bone Miner Res 2014;29:338–47. 23821519 10.1002/jbmr.2032

[50] Ell B , Mercatali L , Ibrahim T , et al. Tumor‐induced osteoclast miRNA changes as regulators and biomarkers of osteolytic bone metastasis. Cancer Cell. 2013;24:542–56. 24135284 10.1016/j.ccr.2013.09.008 PMC3832956

[51] Liu J , Li D , Dang L , et al. Osteoclastic miR‐214 targets TRAF3 to contribute to osteolytic bone metastasis of breast cancer. Sci Rep. 2017;7:40487. 28071724 10.1038/srep40487 PMC5223164

[52] Zhao C , Sun W , Zhang P , et al. miR‐214 promotes osteoclastogenesis by targeting Pten/PI3k/Akt pathway. RNA Biol. 2015;12:343–53. 25826666 10.1080/15476286.2015.1017205 PMC4615895

[53] Krzeszinski JY , Wei W , Huynh H , et al. miR‐34a blocks osteoporosis and bone metastasis by inhibiting osteoclastogenesis and Tgif2. Nature. 2014;512(7515):431–5. 25043055 10.1038/nature13375 PMC4149606

[54] Kobayashi K , Takahashi N , Jimi E , et al. Tumor necrosis factor alpha stimulates osteoclast differentiation by a mechanism independent of the ODF/RANKL‐RANK interaction. J Exp Med 2000;191:275–86. 10637272 10.1084/jem.191.2.275 PMC2195746

[55] Zhao B , Grimes SN , Li S , Hu X , Ivashkiv LB . TNF‐induced osteoclastogenesis and inflammatory bone resorption are inhibited by transcription factor RBP‐. J. J Exp Med. 2012;209:319–34. 10.1084/jem.20111566 PMC3280875 22249448

[56] Miller CH , Smith SM , Elguindy M , et al. RBP‐J‐Regulated miR‐182 promotes TNF‐alpha‐induced osteoclastogenesis. J Immunol. 2016;196:4977–86. 27183593 10.4049/jimmunol.1502044 PMC4893988

[57] Bonewald LF . The amazing osteocyte. J Bone Miner Res 2011;26:229–38. 21254230 10.1002/jbmr.320 PMC3179345

[58] Zeng HC , Bae Y , Dawson BC , et al. MicroRNA miR‐23a cluster promotes osteocyte differentiation by regulating TGF‐beta signalling in osteoblasts. Nat Commun. 2017;8:15000. 28397831 10.1038/ncomms15000 PMC5394267

[59] Davis HM , Pacheco‐Costa R , Atkinson EG , et al. Disruption of the Cx43/miR21 pathway leads to osteocyte apoptosis and increased osteoclastogenesis with aging. Aging Cell. 2017;16:551–63. 28317237 10.1111/acel.12586 PMC5418188

[60] Fu J , Hao L , Tian Y , Liu Y , Gu Y , Wu J . miR‐199a‐3p is involved in estrogen‐mediated autophagy through the IGF‐1/mTOR pathway in osteocyte‐like MLO‐Y4 cells. J Cell Physiol 2018;233:2292–303. 28708244 10.1002/jcp.26101

[61] Valadi H , Ekström K , Bossios A , Sjöstrand M , Lee JJ , Lötvall JO . Exosome‐mediated transfer of mRNAs and microRNAs is a novel mechanism of genetic exchange between cells. Nat Cell Biol 2007;9:654–9. 17486113 10.1038/ncb1596

[62] Braicu C , Tomuleasa C , Monroig P , Cucuianu A , Berindan‐Neagoe I , Calin GA . Exosomes as divine messengers: are they the Hermes of modern molecular oncology? Cell Death Differ 2015;22:34–45. 25236394 10.1038/cdd.2014.130 PMC4262777

[63] Liu J , Li D , Wu X , Dang L , Lu A , Zhang G . Bone‐derived exosomes. Curr Opin Pharmacol 2017;34:64–9. 28881252 10.1016/j.coph.2017.08.008

[64] Xie Y , Chen Y , Zhang L , Ge W , Tang P . The roles of bone‐derived exosomes and exosomal microRNAs in regulating bone remodelling. J Cell Mol Med 2017;21:1033–41. 27878944 10.1111/jcmm.13039 PMC5387131

[65] Li D , Liu J , Guo B , et al. Osteoclast‐derived exosomal miR‐214‐3p inhibits osteoblastic bone formation. Nat Commun. 2016;7:10872. 26947250 10.1038/ncomms10872 PMC4786676

[66] Sun W , Zhao C , Li Y , et al. Osteoclast‐derived microRNA‐containing exosomes selectively inhibit osteoblast activity. Cell Discov. 2016;2:16015. 27462462 10.1038/celldisc.2016.15 PMC4886818

[67] Cui Y , Luan J , Li H , Zhou X , Han J . Exosomes derived from mineralizing osteoblasts promote ST2 cell osteogenic differentiation by alteration of microRNA expression. FEBS Lett. 2016;590:185–92. 26763102 10.1002/1873-3468.12024

[68] Sato M , Suzuki T , Kawano M , Tamura M . Circulating osteocyte‐derived exosomes contain miRNAs which are enriched in exosomes from MLO‐Y4 cells. Biomed Rep. 2017;6:223–31. 28357077 10.3892/br.2016.824 PMC5351302

[69] Qin Y , Peng Y , Zhao W , et al. Myostatin inhibits osteoblastic differentiation by suppressing osteocyte‐derived exosomal microRNA‐218: a novel mechanism in muscle‐bone communication. J Biol Chem 2017;292:11021–33. 28465350 10.1074/jbc.M116.770941 PMC5491785

[70] Filipowicz W , Bhattacharyya SN , Sonenberg N . Mechanisms of post‐transcriptional regulation by microRNAs: are the answers in sight? Nat Rev Genet 2008;9:102–14. 18197166 10.1038/nrg2290

[71] Lian JB , Stein GS , van Wijnen AJ , et al. MicroRNA control of bone formation and homeostasis. Nat Rev Endocrinol 2012;8:212–27. 22290358 10.1038/nrendo.2011.234 PMC3589914

